# COVID-19 vaccination: challenges in the pediatric population

**DOI:** 10.3389/fpubh.2024.1390951

**Published:** 2025-01-29

**Authors:** Alice Nicoleta Azoicai, Ingrith Miron, Ancuta Lupu, Monica Mihaela Alexoae, Iuliana Magdalena Starcea, Mirabela Alecsa, Vasile Valeriu Lupu, Ciprian Danielescu, Alin Horatiu Nedelcu, Delia Lidia Salaru, Felicia Dragan, Ileana Ioniuc

**Affiliations:** ^1^Pediatrics, “Grigore T. Popa” University of Medicine and Pharmacy, Iasi, Romania; ^2^Faculty of Medicine, “Grigore T. Popa” University of Medicine and Pharmacy, Iasi, Romania; ^3^Faculty of Medicine and Pharmacy, University of Oradea, Oradea, Romania

**Keywords:** vaccine, COVID-19, SARS-CoV-2, efficacy, safety, children

## Abstract

Vaccination is considered to be one of the most effective means of protecting individuals and populations from the risks associated with exposure to various pathogens. The COVID-19 pandemic, caused by the severe acute respiratory syndrome coronavirus 2 (SARS-CoV-2), affected people of all ages worldwide. In response, several pharmaceutical companies rapidly leveraged their resources to develop vaccines within a very short period of time, leading to the introduction of new, improved, and combination vaccines for community-wide immunization. This review aims to provide a summary of the available literature on the efficacy and safety of COVID-19 vaccines in the pediatric population ranging from 0 to 18 years. An analysis of recent published studies reveals that the majority of clinical trials have reported a sustained immune response following COVID-19 vaccination in children across various age groups worldwide. The majority of the authors highlighted the effectiveness and safety of immunization schedules in children and adolescents. The population-level efficacy of this vaccination remains to be determined, provided that the benefits outweigh the potential risks. Long-term side effects must still be monitored to enable the development of safer and more effective vaccines for future pandemics.

## 1 Introduction

The COVID-19 infection, caused by the severe acute respiratory syndrome coronavirus 2 (SARS-CoV-2), emerged at the end of 2019 in Wuhan, China, and rapidly spread to all continents. This virus affects people of all ages worldwide and was declared a pandemic by the World Health Organization (WHO) on 11 March 2020 (1). The global impact of COVID-19, with hundreds of millions of confirmed cases and over 9 million fatalities, has spurred significant scientific interest. Researchers have focused on understanding the pathogenesis of the disease, its epidemiology, and how it varies with age or pre-existing clinical conditions. There is also a strong emphasis on exploring methods of prevention and treatment.

Vaccination is considered to be one of the most effective interventions for individual and collective protection of the population against the risks caused by exposure to various pathogens. Vaccination efforts at local, regional, national, and global levels have consistently demonstrated their benefits over time, eradicating life-threatening diseases, reducing morbidity, and limiting the consequences of infections that determined suffering, disability, and death in the pre-vaccine era (such as diphtheria, tetanus, whooping cough, poliomyelitis, measles, rubella, and so on). The proof of these effects is also represented by the fact that the number of deaths caused by vaccine-preventable diseases decreased from 0.9 million in 2000 to 0.4 million cases reported in 2010 (2, 3).

A key benefit of vaccination programs is the induction of population-wide immunity, often referred to as “herd immunity.” This immunity protects the community against disease through widespread vaccination, resulting in a decrease in pathogen circulation within that community (3). Among various medical interventions involving biologically active medications, the protection of an entire community is uniquely achievable through extensive vaccination efforts (4, 5). Unvaccinated individuals can benefit from “herd immunity,” which creates a potential ethical issue of “free-riders.” These are people who gain the advantages of vaccination programs without personally taking on any of the risks associated with receiving the vaccine directly (3, 4).

The lack of high-quality research hampers a comprehensive understanding of the post-acute and long-term consequences of COVID-19. By standardizing the definitions and harmonizing research, diagnosis, and treatment approaches for long-term COVID-19, we can improve the coherent collection of national and international data. This would enable better estimates of incidence, prevalence, and risk factors tailored to different age groups. There is a critical need for large, coordinated longitudinal studies to explore the various aftereffects of SARS-CoV-2 infection in children and adolescents. While relatively few studies have targeted this demographic, patient support groups have reported that many children suffer from the lingering effects of COVID-19. High-quality evidence is urgently needed, and this could be facilitated by conducting controlled trials that account for societal variables.

Additionally, robust case–control studies are essential for identifying sources and risk factors for various long-term COVID-19 conditions, which will aid in the development of targeted interventions and support mechanisms.

## 2 Methods

A substantial body of literature has emerged on surveillance advancements during the COVID-19 pandemic. While wastewater epidemiology has seen extensive research, topics such as health equity for racial and ethnic minorities are less studied. In areas with extensive research, conducting systematic reviews may be the logical next step. Conversely, in fields where knowledge is scarce, further research is essential to advance monitoring in the post-pandemic era.

Additionally, the widespread implementation of these surveillance techniques necessitates a comprehensive analysis of potential consequences, including ethical, legal, security, and equity implications, as highlighted by numerous studies (5). Our literature search was conducted using Medline and Medscape, focusing on articles published from 2019 to 2024 with keywords including “pandemic,” “SARS-COV2 infection,” “vaccine,” “children,” “safety,” and “efficacy.”

Researchers’ findings support the development of multidisciplinary collaborative rehabilitation programs for younger populations impacted by COVID-19 and the deployment of monitoring systems to monitor the health effects of the virus. There are meta-analyzes, cross-sectional studies, reviews, and prospective studies to prove that vaccination in early age groups can reduce the burden of COVID-19 infection. To close the gap between research results and clinical application in this discipline, it is critical that non-physical outcomes be given top priority in future attempts (6).

Our objective is to offer suggestions for filling in the knowledge gaps on the long-term effects of COVID-19 on children. Priorities for studying the effects of COVID-19 on children’s bodies, minds, emotions, and social interactions must be determined within a systems framework and coordinated on a national and worldwide scale. We call on national and international funding organizations to promote coordination efforts between families impacted by long-term COVID-19 and experts such as pediatricians, epidemiologists, rehabilitation clinicians, psychologists, psychiatrists, researchers, and public health experts. A dynamic assessment of the effects of prolonged COVID and the care required for children with this illness may be made easier by longitudinal repeated examinations of representative samples of children and adolescents with a diagnosis of SARS-CoV-2 infection and matched control individuals. This type of research design could also help clinicians to discriminate between short- and longer-term outcomes of the condition and the impact, as well as provide evidence-based profiles of individuals who are affected by long-term COVID-19, identify those at higher risk, and inform targeted interventions to improve long-term outcomes.

Children may serve as a reservoir for the virus and spread it, even if the majority of them are asymptomatic or just mildly afflicted by COVID-19 infections. The financial burden and vaccine accessibility are crucial factors in requiring the COVID-19 vaccine. Before vaccinations are required, a number of ethical issues also need to be considered. Unknown are the vaccine’s efficacy and safety for kids, their vulnerability to infection, their part in the disease’s spread, and the anticipated advantages. Moreover, religious beliefs, parental hesitancy, media involvement, and anti-vaccination campaigns might also be considered real challenges in children’s COVID-19 vaccination.

## 3 COVID-19 vaccines

This infection is characterized by clinical and evolutionary polymorphism, which is influenced by the viral variants that emerge over time (such as the alpha, delta, and omicron strains) and the age at which the infection occurs. This variability contributes to skepticism regarding the vaccination of children (1). This situation is principally based on the limited knowledge about advancements in developing more effective and less harmful vaccines, and this is again a reason for which authors should focus on proving the efficacy of vaccines and the lack of side effects. Then, there is the deep-rooted idea that the best immunization is provided by the disease. Therefore, the human body should be allowed to face the disease (7), a principle that has still not been proved in the case of COVID-19 infection.

For example, the pediatric population evaluated in studies and meta-analyzes is inferior to cohorts of adult subjects, which implies a greater degree of extrapolation of the obtained data but also necessitates continuous efforts. The lack of studies focusing on the efficacy and safety of COVID-19 vaccines for children and infants complicates efforts to vaccinate these population groups. While COVID-19 vaccination in adults was reported to decrease in percentage, children and young people (CYP) registered higher rates of vaccination in the past 4 years (8).

The main benefits of COVID-19 vaccination for children include overcoming potential side effects and achieving immunity. Nevertheless, even minor vaccination risks need to be considered, as the likelihood of serious illness in otherwise healthy children is very low. The majority of the potential benefit of vaccination in preventing serious illness and/or PIMS-TS/MIS-C has been diminished because of pre-existing immunity to infection and decreased incidence of hyper-inflammatory response as a result of both viral evolution and pre-existing immunity. Any possible advantage in stopping the spread of viruses is negligible and transient. If there is already a high level of community immunity due to infection, then any benefits from temporarily boosted immunity for otherwise healthy children may be outweighed by the high financial and opportunity costs associated with starting new vaccination programs. For children with significant comorbidities, there is a much larger absolute reduction in risk provided by periodic vaccination, which is the basis of the majority of current national public health recommendations (9).

Possible (or probable) post-vaccination reactions, in the context of the use of biologically active vaccines, are currently reduced as a result of the evolution of knowledge in the field of modern vaccinology. The security measures adopted in the case of the production and use of vaccines, as in fact of any procedure or medicinal product that is applied to an individual or a large population, provided safety for the recipient. Developing vaccines with high immunogenicity and low reactogenicity characteristics has determined an extremely limited possibility of installing such reactions under the condition of compliance with specific regulations and protocols, which are necessary in the case of application of preventive or therapeutic action (10, 11).

The increasingly advanced knowledge of the mechanisms of the vaccines, as well as the circumstances that allow the minimization of risks, is a priority for the medical world that has the duty to make known these scientific truths in order to regain the trust of the population in a measure that has demonstrated, over time, to be beneficial to the individual and the human community (8, 12). There has to be higher compliance from the caregivers (parents, family doctors, specialists) in order to protect young patients against COVID-19 infection, which proved to be life-threatening in children’s pathology (13).

The majority of the studies initially stated that there is a low susceptibility to SARS-CoV-2 infection in children. The disease also has a generally milder course than in adults, with a low percentage of severe cases and usually burdened by an underlying chronic pathology (chronic pulmonary conditions such as cystic fibrosis, tuberculosis, pulmonary malformations, ciliary dyskinesia, cardiovascular malformations, genetic syndromes, oncological, and renal diseases) (13, 14). The phenomenon could be explained by several mechanisms. One would be the action of the innate immune response, the first line of defense against pathogens, which tends to be more active in children. Paradoxically, another explanation could be the immaturity of the children’s immune system, which is probably not able to sustain the cytokine storm similar to that observed in the adult population. Also, the different distribution of membrane ACE2 receptors in adults and children with a lower receptor binding capacity could be responsible for the attenuated symptoms in their case, as well as a higher plasma concentration of soluble ACE2 receptors, the particular interaction with these receptors, thus being able to limit their replication in tissues (15, 16).

Multiple trials have evaluated the efficacy and safety of COVID-19 vaccines in both healthy adults and patients with comorbidities (14–19). Similarly, vaccination against coronavirus can prevent serious outcomes or hospitalization following the natural infection (20). Of note, children and adolescents had their education, safety, and mental and physical wellness negatively affected during the pandemic, making vaccination crucial for them to avoid further isolation (21). All children and adolescents should be considered for COVID-19 vaccination for their own protection against the infection and its different outcomes, and more importantly, because they are part of the COVID transmission cycle, thus being carriers and serving as a reservoir of disease for elders (parents, grandparents) (8–12, 22–24).

Several clinical trials supported the favorable immune response, effectiveness, and safety profiles of COVID-19 vaccines in healthy children and adolescents and even in those with underlying medical conditions (25–28). In almost all studies, authors aimed to collect data regarding the immunogenicity, efficacy, and safety of COVID-19 vaccines to guide healthcare workers and families in vaccinating the younger population.

Patients with autoimmune diseases or immunodeficiencies have a higher risk of COVID-19 infections, hospitalization, and death than the general population and are a priority for vaccination (29). Due to a lack of information, medication side effects, and the possibility of triggering severe side effects in those special categories of patients, both doctors and caregivers are often reserved in recommending and/or accepting COVID-19 immunization.

Juvenile idiopathic arthritis (JIA) is the most common pediatric rheumatic disease, the burden within young children and adolescents being related to infectious risk factors and autoimmunity as a trigger. This is the reason that makes preventing viral infections the most effective tool in controlling the disease. Authors have been challenged in proving the efficacy and the real need for COVID-19 vaccination for those specific population categories. An observational study that compares the immunogenicity and the safety of the Pfizer COVID-19 vaccine in patients with JIA in the age group between 12 and 16 years and a group of healthy controls shows no statistically significant differences in the average levels of antibodies in the patients and controls, in line with other studies of Pfizer immunogenicity in adolescents with JIA. An important matter is that of immunosuppressive therapy, and this is why methotrexate was discontinued during the weeks of the first and second vaccine inoculations. Non-steroidal anti-inflammatory drugs (NSAIDs) and biological drugs were not discontinued while treating the patients for COVID-19 (30). The authors also observed that patients with systemic JIA produced lower antibody titers than patients with other types of JIA (31). It’s been underlined in those findings the fact that COVID-19 vaccination does not interfere with the JIA treatment and does not exacerbate symptoms of the disease. Authors have proven, in fact, that vaccination protects against developing COVID-19 in children with JIA (32).

Since the beginning of the pandemic, children with primary immune deficiency (PID) have been the main category of concern (33). Before the worldwide extension of the viral strains of COVID-19, children with primary immune deficiencies were also at very high risk of acquiring and manifesting infections, making them a special category of eligible candidates for the majority of the vaccines. Transplantation, substitutive therapy, specific medication, young age, and comorbidities were the main concerns in having the PID children vaccinated against COVID-19. Questions were raised regarding the benefits or the risks for those special patients. Although PID is among the main preexisting conditions associated with COVID-19 infection in children, patients with phagocytic or antibody defects or children with combined PID who have already been transplanted can develop mostly asymptomatic or mild COVID-19 (34, 35). The authors agreed on the need for pediatric patients with primary immune deficiency to be vaccinated, thus reducing the risks of severe COVID-19 illness and death. This most vulnerable population must be sheltered from infection, taking into consideration that the immune response to SARS-CoV-2 vaccines may differ in people with primary immune deficiency. This is why an individual approach is required, and specific organizations, such as the Centers for Disease Control and Prevention (CDC), have developed specific guidance, COVID-19 vaccination being the primary prevention strategy (36), along with specific and reliable therapies that have been approved in the case of those patients.

PID pediatric patients may also develop prolonged or severe forms of COVID-19 infection, and it is mandatory to define their immune response to the disease. Thus, the Committee of Experts on Primary Immunodeficiency has included vaccination both as a diagnostic tool (to assess the specific antibody response to protein and polysaccharide antigens) and as a means of prevention (37). The response to COVID-19 infection by developing antibodies was assessed later on, and the efficacy of vaccination relied on the detection of specific antibodies against SARS-CoV-2 antigens. In the general population, the level of neutralizing antibodies is correlated to protection, and mRNA vaccination generated robust humoral and cellular immune memory to SARS-CoV-2 for at least 6 months following mRNA vaccination (32). In particular, patients with PID may not be able to maintain this immunogenicity over time. However, even in healthy individuals, the antibody response may wane over time or may not be detectable in patients with antibody deficiency (37).

For children and adolescents with allergic conditions such as wheezing and asthma, there were concerns regarding the safety of vaccination, given the risk of having an anaphylactic reaction to a COVID-19 vaccine, even though severe allergic conditions were not noted in a pediatric population. A systematic review of the literature noted that the incidence of an allergic reaction to an mRNA-based COVID-19 vaccine is 7.91 cases per million doses (95% CI 4·02–15·59) (40), a very low risk if we take into consideration the benefit of protection. There were no reported anaphylactic fatalities related to COVID-19 vaccination, and the local allergic reactions resolved rapidly without long-term sequelae. Furthermore, revaccination after an initial allergic reaction was well tolerated within those patients (41).

Anaphylaxis is unpredictable, so a prudent approach is advisable, such as allergic evaluation in case of previous systemic reactions to vaccines or drugs. Risk assessment of allergic reactions to COVID-19 vaccines is useful in limiting contraindications to vaccination and obtaining medical recommendations and parental consent. All vaccine centers should follow international and national guidelines, and doctors should be trained in preventing, recognizing, and managing post-vaccinal anaphylaxis (42).

## 4 Immunogenicity of COVID-19 vaccines in children

Immunogenicity concerns regarding children, including those with chronic illnesses as well as for healthy individuals, have been in focus since the beginning of the pandemic. The primary concern was whether the immunogenicity achieved with one or multiple vaccine doses varies significantly based on age, medical history, or immune response in children. Specialists must consider factors such as age group, immune status, comorbidities, chronic illnesses, and/or immunosuppressive conditions. It can be stated that there still is an urgent need for continuous surveillance and extensive studies to assess the real status of immunogenicity achieved with vaccination versus naturally acquired antibodies (43). The differences between the population groups that were observed in extensive studies can explain the lack of protection against further infection in some categories of individuals with one or multiple vaccine protections (such as in the case of immune-deficient children).

Authors reported approximately 99% serologic response to the mRNA-1273 Moderna vaccine in people aged 12–17 years old, compared to a 98.6% response in younger adults—according to Ali et al. (44). Furthermore, the findings stated that the neutralizing antibody titers in younger ages (children) showed no inferiority when compared to those in older patients.

Frenck et al. (45) conducted a randomized clinical trial to assess the effects of the BNT162b2 (Pfizer) vaccine in children and adolescents aged 12–15 years. The authors found these subjects developed higher post-vaccination antibody titers compared to vaccinated younger adults and the control group. Other authors (46, 47) revealed that nearly all (99.2%) of Pfizer-vaccinated children aged 5–11 years achieved a satisfactory serologic response 1 month after receiving the second dose.

These findings support the notion that immunization should be considered for early age groups, as many studies suggest that younger children tend to produce higher rates of antibody production. This may be due to the innate immune system, which is more active in infants and young children, enabling them to develop higher titers of antibodies and maintain these at protective levels for extended periods. However, the paucity of extensive studies confirming the safety of vaccinations in these age groups remains a concern, often due to parental hesitancy to provide consent.

## 5 Efficacy of COVID-19 vaccines in children and adolescents

The benefit of immunization was demonstrated in the adult population, as the levels of morbidity and mortality due to COVID-19 infection dramatically decreased worldwide. Regarding passive immunization in young children, there is still controversy among authors who conducted studies centered on the real need for vaccinating children. The majority of the studies initially stated that there is a low susceptibility to SARS-CoV-2 infection in children, the disease also having a generally milder course than in adults, with a low percentage of severe cases and usually burdened by an underlying chronic pathology (48).

On the other hand, several studies showed the need for children and adolescents’ COVID-19 vaccination—first for the protection against the infection and second because they are part of the COVID-19 transmission cycle. Children represent important carriers of the disease, regardless of the fact that they express the symptoms more or less prominently, thus serving as a reservoir of disease for elders, in which the outcome may be fatal. Isolation, lack of socialization methods, and mental and behavioral changes within the pandemic were issues that conducted authors in providing the population with “pro” and “con “arguments regarding the efficacy of vaccination in children and adolescents and the long-term protection against the infection.

The efficacy of the COVID-19 vaccine in children aged 5–11 years was reported to be nearly 91% after the second dose, according to Frenck et al. (45), using the Pfizer vaccine. Moreover, the authors noted a remarkable efficacy rate of 100% in individuals aged 12–15 years (45). In another study assessing the efficacy of the Pfizer vaccine in adolescents aged 12–18 years, only two patients out of 57 participants contracted COVID-19 after being immunized: one patient tested positive before receiving the second dose, and the other 46 days post-second dose (46).

A particular group of potential vaccine recipients—those with underlying medical conditions, chronic illnesses, or immunodeficiency due to chemotherapy regimens, as well as children with innate immunodeficiencies—requires careful evaluation of vaccine efficacy. The beneficial effects on these children and adolescents have been assessed in studies encompassing multiple vaccine types and considering various age groups.

Adolescent patients with solid tumor malignancies who completed the full Pfizer vaccine immunization schedule were not found to be at risk of developing COVID-19 infection (41). In studies involving other vaccine types eligible for the population under 21 years of age, such as Moderna, CoronaVac, and ZyCov-D, efficacy rates of 93.3, 65.5, and 100% protection against COVID-19 infection were reported among participants aged 12–19 years, respectively (46). Further extensive studies on additional vaccine types, including Sinopharm and COVAXIN (NCT04918797), also suggested high protection efficacy against COVID-19 in the 2–18-year-old age group (46).

There is also the question of whether efficacy should be discussed in terms of age group, as long as innate immunity may be an advantage in obtaining higher levels of protective antibodies in young children.

Recently, a group of Italian authors conducted a retrospective population study, assessing vaccine efficacy against SARS-CoV-2 infection and the severe COVID-19 infection rates (defined as an infection leading to hospitalization or lethal outcome) by linking the national COVID-19 surveillance system and the national vaccination registry. All Italian children aged 5–11 years without a previous diagnosis of infection were eligible for inclusion. The authors followed up with the patients over a 4-month period of time, relying on unvaccinated children as the reference group. Furthermore, the authors estimated the vaccine efficacy in those participants who were partly vaccinated (one dose) and in those who were fully vaccinated (two doses) (47).

The results showed that 35.8% of children aged 5–11 years included in the study had received two doses of the vaccine, and only 4.5% had received only one dose; 59.6% of all age groups represented the children who were unvaccinated. The results were not promising, with multiple cases of severe COVID-19 (627 hospitalizations, 15 admissions to intensive care units, and two deaths), as well as many mild infections. Overall, authors assessed the vaccine efficacy in the fully vaccinated group as being only 29.4% against SARS-CoV-2 infection and not higher than 411% against severe COVID-19, whereas vaccine efficacy in the partly vaccinated group was rather similar, with 27.4% efficacy against SARS-CoV-2 infection and 38.1% against severe COVID-19 (47). To sum up, the results demonstrated that vaccination against COVID-19 in children aged 5–11 years in Italy had, in fact, lower effectiveness in preventing SARS-CoV-2 infection and severe COVID-19 than in individuals aged 12 years and older. Effectiveness against infection appears to increase up to 14 days following immunization, with a decrease after completion of the current primary vaccination cycle of 43–84 days (47).

## 6 Safety of COVID-19 vaccines in children and adolescents

Regarding the safety and security of all vaccines, there is a comprehensive and lengthy chain of surveillance measures and regulations established in each region or country. Initially, it is determined whether the new vaccine can undergo evaluations to receive the license. The special accredited committees for the supervision and licensing of a vaccine, in collaboration with the manufacturers, monitor the safety and efficacy of the vaccine through a strategy based on national or international laws and regulations.

European regulation on the authorization and population use of medicinal devices for human use includes vaccines among immunological biological products. The evaluation of a vaccine is carried out identically to that of any medicine. The stages are laborious and take a long time to be carried out. They are completed by drawing up documentation that includes the results of clinical and pharmaceutical studies, particularly those related to the product’s safety.

Improved vaccine safety monitoring and the timely, accurate, and transparent disclosure of safety findings were crucial aspects of the COVID-19 response during the US COVID-19 pandemic immunization program. This comprehensive approach included clinical consultations, long-term follow-up on individual cases of myocarditis after immunization, both active and passive surveillance, and monitoring of pregnancy and infant outcomes. The most efficient methods for disseminating the latest information to stakeholders and the public involved updating agency websites, engaging through social media, presenting findings to federal advisory bodies, and publishing safety results in scientific journals (48, 49).

Safety studies have been conducted for vaccines that have been approved for years and decades, thus guaranteeing the possibility of long-term surveillance of subjects. The COVID-19 pandemic was the turning point in drawing a new era for “fast-forward” developing and testing vaccines. A key point considered to be crucial for controlling the virus transmission and pandemic annihilation was the possibility of initializing vaccine development studies. This was the reason for observing and assessing early side effects even at the same time as actual immunization and not waiting longer for outcomes *in vitro* studies. Several pharmaceutical companies had the opportunity and the industrial means to develop a vaccine quickly, releasing new, improved, and combined vaccines for community immunization (50, 51).

Reported adverse reactions were mild to moderate and self-limiting, as long as the current studies have shown a significant percentage of parents willing to vaccinate their children and adolescents against the new coronavirus. The most common adverse reactions following immunization comprised injection site pain and erythema, headache, fatigue, fever, and chills (52–54), nothing more than in the case of other studied vaccines.

The authors had the opportunity to assess the side effects in a specific and distinct group within the community. In the case of adolescents and young adults (aged 16–25 years) residing in a long-term care facility who received the Pfizer vaccine, 84% experienced mild adverse reactions after the first dose, and 74.2% reported similar effects following the second dose. These reactions included discomfort, nausea/emesis, diarrhea, fever, chills, headache, and skin erythema at the inoculation site (54).

The Pfizer vaccine was administered to pediatric patients and young adults with juvenile inflammatory arthritis (JIA) aged 16–21 years, with no reported exacerbation of the chronic disease, indicating a good safety profile for this particular group (54). However, transient increases in agitation and changes in seizure patterns, specifically cluster seizures, were observed in recipients aged 12–15 years old with underlying neurologic and mental conditions. These observations highlight the need for further monitoring of post-immunization side effects in these vulnerable groups (53, 54).

Recent extensive studies have reported an increased incidence of myocarditis and pericarditis after COVID-19 vaccination, particularly among male adolescents and young adults, raising major global concerns. For instance, in Israel, five male patients with a median age of 23 developed myocarditis after receiving the BNT162b2 vaccine (55). Additionally, in the United States, eight male adolescents presented with myocarditis within 4 days of receiving a dose of the BNT162b2 vaccine, as noted by the authors (56). Another report highlighted a series of 25 children aged 12–18 years diagnosed with probable myocarditis after COVID-19 mRNA vaccination at eight US centers between May and June 2021. These cases did not show any clinical or functional impact post-treatment. Treatment approaches varied: three cases were managed with non-steroidal anti-inflammatory drugs, while four patients received a combination of intravenous immunoglobulin and cortisone therapy to control the condition (57).

Recent reports have demonstrated that multisystem inflammatory syndrome (MIS) can occur after SARS-CoV-2 vaccination, now identified as “MIS-V” rather than “MIS-V.” An instance of such symptoms was documented in an 18-year-old adolescent following the administration of the Pfizer-BioNTech BNT162b2 vaccine (58). The primary clinical features mirrored those observed during the acute phase of infection, including fever lasting for 3 consecutive days, mild to moderate pericardial effusion, elevated levels of CRP, NT-BNP, troponin T, and D-dimers, which is evidence of cardiac involvement, and positive IgG SARS-CoV-2 antibodies, which helps to establish a link between the vaccination and the observed symptoms (58).

## 7 COVID-19 vaccination in MIS-C patients

Multisystem inflammatory syndrome developed after COVID-19 infection represents a milestone for developing further medication and prophylactic therapy, both for adults and especially for children, in which the outcome was severe (even lethal in some cases). Study data regarding adverse reactions after COVID-19 vaccination in adult pediatric patients with a history of multisystem inflammatory syndrome (MIS-C) are limited. This lack of safety and efficacy data in this specific population may cause limited approval for vaccination from healthcare professionals and hesitancy and concern for caregivers and parents. There is an interest in applying most of the study designs to a wide population of children when the analysis design and the reported data’s applicability can be extended. Therefore, assessing the results and conclusions would appear to be more trustworthy.

MUSIC is a multicenter, cross-sectional study including 22 North American centers participating in a National Heart, Lung, and Blood Institute, National Institutes of Health-sponsored study, Long-Term Outcomes After the Multisystem Inflammatory Syndrome in Children. The pediatric population with a prior diagnosis of MIS-C that appeared to be eligible for COVID-19 vaccination at the time of enrolling (age ≥ 5 years; ≥90 days after MIS-C diagnosis) were surveyed over a period of 3 months regarding COVID-19 vaccination status and reported adverse reactions (59). The authors were trying to assess whether MIS-C would be a condition to take into consideration when establishing the need, the benefit, or the actual risk for vaccination. Patients were also randomized based on age group, ethnicity, and medication intake.

Almost half of all the 385 vaccine-eligible patients surveyed, 185 (48.1%), received at least one vaccine dose; the majority of vaccinated patients (73.5%) were male, at a median age of immunization of 12 years. Among vaccinated patients, there were mostly white children, as well as a significant percentage of Asian, Hispanic, and Black ethnicity. The median time lapse from the initial moment of MIS-C diagnosis to the first vaccine dose inoculation was almost 9 months. Out of them, 31 patients (16.8%) received one vaccine dose, 142 (76.8%) received two doses, and 12 (6.5%) received all three doses of the vaccine. It is important to observe that almost all patients received the BNT162b2 vaccine—98.9% (59).

Minor adverse reactions were observed in almost half of the study group—48.6%. The complaints most often included arm soreness and/or fatigue, which did not require medical attention. However, in 32 patients (17.3%), adverse reactions were treated with medications, most commonly for the fever and the pain, using either acetaminophen or ibuprofen. Only four patients were addressed for medical evaluation, but none required testing or hospitalization. Moreover, neither of the patients included in the study developed an MIS-C symptomatology after vaccination nor cardiovascular events, which are a key point in assessing the safety of immunization in young children (59).

The authors did not report any patients with serious adverse events, such as myocarditis or recurrence of MIS-C (59), proving that there were no severe adverse events after COVID-19 vaccination. Findings suggest that the safety profile of COVID-19 vaccination administered at a time-lapse of at least 90 days following MIS-C appears to be similar to that assumed in the general population.

Zambrano et al. (60) compared the odds of being fully vaccinated with two doses of the BNT162b2 vaccine (≥28 days before hospital admission) between MIS-C case patients and hospital-based controls who tested negative for SARS-CoV-2. Authors examined those associations by age group, timing of vaccination, and periods of Delta and Omicron variant predominance (60). This study was conducted across 29 hospitals in 22 US states in the Centers for Disease Control and Prevention (CDC)–funded Overcoming COVID-19 (OC-19) pediatric vaccine effectiveness network. Clinical outcomes among MIS-C patients for those requiring ICU admission, vasopressor support, and noninvasive or invasive mechanical ventilation were clearly in favor of those who received a complete vaccination schedule. Those findings are also supported by a comparison of MIS-C cases resulting in life support or death between vaccinated and unvaccinated patients.

In comparison, Cortese et al.., out of a cohort of 77 patients, 58 children were identified who developed MIS-C within 90 days after receiving a COVID-19 vaccine and had evidence of past or recent SARS-CoV-2 infection. Additionally, four children met the MIS-C criteria but had no evidence of SARS-CoV-2 infection. The authors were unable to conclusively determine whether the COVID-19 vaccination contributed to the MIS-C cases identified in the study group. This uncertainty was partly due to the expectation of an increase in MIS-C cases associated with the Omicron variant of SARS-CoV-2, which coincided with the availability of the COVID-19 vaccine for this age group approximately 5–6 weeks prior to the enrollment of cases in the study (61).

Table 1 summarizes the studies regarding the efficacy and safety of vaccination in children.

**Table 1:** Current studies recommendations and evidence regarding the safety and efficacy of mRNA COVID-19 vaccines.

Authors	Outline	No. patients	Efficacy/safety	Age range	Country/region
Opoka-Winiarska et al. (32)	Children and adolescents with JIA with remission without treatment or on long-term treatment—cDMARDs or even bDMARDs, can be safely vaccinated for COVID-19	43 with JIA	++/++	0–18 years	Poland
Quinti et al. (37)	Despite the antibody deficiency, T-cell immunity is thought to be largely intact in many patients with CVID, as immunologists recommend routine administration of multiple vaccines, including COVID-19 immunization	9 with PID	+/+	6–18 years and adult patients	Italy
Krantz et al. (42)	The majority of patients with allergic reactions to mRNA COVID-19 vaccines can safely tolerate a second dose of immunization	159	++/++	0–18 years	Australia
Sacco et al. (47)	Vaccine efficacy was 31% (95% CI 9–48) at 14–82 days after completion of the primary cycle in a sample of 1,364 children aged 5–11 years, very similar to our estimate of 29.4% after a similar interval of 0–84 days after full vaccination	1,364	+/+	5–11 years	Italy
Myers et al. (49)	V-safe contributed to the CDC’s vaccine safety assessments for FDA-authorized COVID-19 vaccines by enabling near real-time reporting of the reactogenicity of the vaccines	9,342,582	++/++	0–18 years	United States
Zambrano et al. (60)	Vaccination with two doses of vaccine is associated with reduced risk of MISC C in children	304	++/++	5–18 years	United States
Cortese et al. (61)	MISC C illness in children after COVID-19 vaccination was below 1/million vaccinated children	58	++/++	0–18 years	
Tartof et al. (66)	BNT162b2 BA.4/5 bivalent mRNA vaccine against a range of COVID-19 outcomes in a large health system in the United States proved effective in a test-negative case–control study	24,246	++/++	0–18 years	United States
Feldstein et al. (67)	Bivalent mRNA COVID-19 vaccines are effective in preventing SARS-CoV-2 infection in children and adolescents aged 5–17 years	2,959	++/++	5–17 years	United States
Aldridge et al. (8)	Uptake of COVID-19 vaccinations among 3,433,483 children and young people showed safety and efficacy in a meta-analysis of UK prospective cohorts	3,433,483	++/++	0–18 years and adult patients	UK
Hu et al. (68)	Ancestral monovalent BNT162b2, mRNA-1273, and NVX-CoV2373 COVID-19 vaccines in US children aged 6 months to 17 years are safe and immunostimulant	410,2016	+/+	6 months-18 years	United States
Buoninfante et al. (69)	Myocarditis associated with COVID-19 vaccination is not as frequent as the first studies outlined at the beginning of the immunization	393	++/++	0–18 years	Italy

Regarding the reason for conducting studies in pediatric age, the majority of the authors state that children’s vaccination against COVID-19 is a moral obligation, as well as a practical need in reducing the burden of the infection, as long as the safety of the vaccines is to be assessed (62). Parental consent is sometimes impaired by the lack of studies in this field. According to the majority of the current literature, our manuscript highlights the crucial importance of children’s vaccination against COVID-19 and the immunogenicity and safety of the vaccines at pediatric age (63).

According to the major topic of this literature review (COVID-19 vaccines, immunogenicity of COVID-19 vaccinations in children, efficacy of COVID-19 vaccines in children and adolescents, safety of COVID-19 vaccines in children and adolescents), the authors created a conceptual table (Table 2) that can be used in the future to produce better, safer, and more effective vaccines for children and adolescents to mitigate the impact of a potential new pandemic (45, 64, 65).

**Table 2:** Vaccine types, efficacy, and side effects in COVID-19 immunization in children.

Vaccine type	Pfizer/BioNTech	Moderna	Novavax
Recommendation	6 m–4 y: 3-dose series ≥5 y: 1-dose	6 m–5 y: 2-dose series ≥6 y: 1-dose	≥12 y: 2-dose series
Efficacy and immunogenicity	75% (6 m–28 m) 71% (2–5 years) 90% (6–11 years) 95% (12–17 years)	51% (6 m–28 m) 36% (2–5 years) 88% (6–11 years) 92% (12–17 years)	No data available No data available No data available 79.5%
Side effects	↑↑ (6 m–28 m) ↑↑ (2–5 years) ↑ (6–11 years) ↑ (12–17 years)	↑ (6 m–28 m) ↑↑ (2–5 years) ↑ (6–11 years) ↑ (12–17 years)	No data available No data available No data available ↑ (12–17 years)
Immunization in MIS-C pediatric patients	No data available	No data available	No data available

## 8 Conclusion

Rapid advancements in research on SARS-CoV-2 infection and COVID-19 immunization have led to recommendations from professional societies affirming the safety and efficacy of vaccinating children and adolescents. The emergence of new variants of SARS-CoV-2 (alpha, delta, omicron) had increased transmissibility and made it clear that acquiring herd immunity would be required to control the pandemic. Coinfection or superinfection comorbidities (viral, bacterial, fungal) equate to a poor prognosis for the pediatric patient. Additionally, younger age groups often exhibit more complex immunological backgrounds, including primary and secondary immunodeficiencies. When vaccinating younger patients, it is crucial to consider the epidemiological context in which acute COVID-19 infection may occur, especially during the seasonal circulation periods of other viral agents such as influenza, parainfluenza viruses, and respiratory syncytial viruses.

The costs associated with pediatric primary care, emergency services, and possible hospital admissions due to severe clinical manifestations, as well as direct or indirect costs for long-term care of children who experience recurrent COVID-19 infections or develop MIS-C, pose a significant economic burden. This burden is substantially higher than the cost of maintaining consistent and comprehensive immunization efforts. Community-wide epidemiological surveillance of COVID-19 infections and immunization in the pediatric population, along with the implementation of specific monitoring protocols, tracking of recurrent hospitalizations due to COVID-19-related respiratory infections, and conducting medium- and long-term follow-up in patients with MIS-C symptoms, will provide crucial data for the implementation of extended prophylaxis.

However, ethical and legal considerations regarding the vaccination of minors cannot be overlooked, particularly in light of ongoing debates in the scientific community about the inclusion of children and young people in COVID-19 vaccine trials. Moreover, it is essential that children, adolescents, and infants are included in comprehensive studies that monitor, describe, and document any adverse reactions following COVID-19 vaccination, especially in patients with a history of MIS-C. These measures are critical to ensuring the safety and efficacy of vaccines for this vulnerable population.

This review highlights that while the population-level effectiveness of this specific vaccination remains to be fully established, the global beneficial response generally outweighs the potential risks. Authors have emphasized the importance of monitoring long-term side effects, as this provides the opportunity to develop newer, safer, and more effective vaccines, potentially including combined formulations, to mitigate the impact of a future pandemic.
